# The Effects of Sharing Awareness Cues in Collaborative Mixed Reality

**DOI:** 10.3389/frobt.2019.00005

**Published:** 2019-02-08

**Authors:** Thammathip Piumsomboon, Arindam Dey, Barrett Ens, Gun Lee, Mark Billinghurst

**Affiliations:** ^1^Empathic Computing Laboratory, University of South Australia, Mawson Lakes, SA, Australia; ^2^School of Product Design, University of Canterbury, Christchurch, New Zealand; ^3^Co-Innovation Group, University of Queensland, Brisbane, QLD, Australia; ^4^Immersive Analytics Lab, Monash University, Melbourne, VIC, Australia

**Keywords:** augmented reality, virtual reality, mixed-space, remote collaboration, awareness cues, user studies, usability, social presence

## Abstract

Augmented and Virtual Reality provide unique capabilities for Mixed Reality collaboration. This paper explores how different combinations of virtual awareness cues can provide users with valuable information about their collaborator's attention and actions. In a user study (*n* = 32, 16 pairs), we compared different combinations of three cues: Field-of-View (FoV) frustum, Eye-gaze ray, and Head-gaze ray against a baseline condition showing only virtual representations of each collaborator's head and hands. Through a collaborative object finding and placing task, the results showed that awareness cues significantly improved user performance, usability, and subjective preferences, with the combination of the FoV frustum and the Head-gaze ray being best. This work establishes the feasibility of room-scale MR collaboration and the utility of providing virtual awareness cues.

## Introduction

One of the main goals of remote collaborative systems is to enable people who are far apart to feel like they are in the same space. Mixed Reality (MR) involves the seamless blending of real and virtual worlds using Augmented Reality (AR) and Virtual Reality (VR) and so provides some unique capabilities to achieve this goal (Billinghurst and Kato, 1999). For example, Augmented Reality (AR) systems can create the illusion that remote people are in the users real space, as 2D video avatars (Kobayashi and Ishii, 1993), virtual characters (Orts-Escolano et al., 2016) or even volumetric video (Zillner et al., 2014; Higuchi et al., 2015; Pejsa et al., 2016). Virtual Reality (VR) systems enable remote people to feel present in the virtual representation of a physical space, using 3D avatars and virtual environment visualization (Otto et al., 2006; Steptoe et al., 2008, 2012). In this research, we compared different combinations of virtual awareness cues to better understand their effects on MR collaboration.

Most collaborative AR and VR systems focus on collaboration between users in either only AR or VR situations. However, there are a few MR collaborative systems that support collaboration between both AR and VR views (Kiyokawa et al., 1999; Billinghurst et al., 2001; Tachi, 2003; Steed et al., 2012). In a similar way, our work explores a scenario where an AR user's local environment is shared remotely with a collaborator through VR. Wearable technologies can now rapidly capture a model of user's surrounding space. Such models can be stored or shared in real time with a remote collaborator, who experiences a reconstruction in VR. In this way, AR and VR users can experience a shared space and collaborate on real-world tasks. One of the closest works to ours is that of Le Chénéchal et al. (2016) who have developed a Mixed Reality system in which an expert user in VR shares viewpoint and gesture cues with an AR user in order to help them complete a real-world task. Similarly, the work of Oda et al. (2015) uses shared gesture and pointing cues between an expert in a VR expert and worker in AR to help with assembly tasks. In contrast, our study adds the use of gaze cues from both AR and VR participants and focuses on room scale collaboration rather than a limited workspace.

There are many possible applications of this type of systems such as emergency response, remote maintenance, education, and others. This scenario extends earlier work by others in using collaborative MR systems for crime scene investigation (Poelman et al., 2012), industrial assembly (Oda et al., 2015) and teaching (Nawahdah and Inoue, 2011). Our research builds on these previous works by implementing virtual communication cues within a room-sized space, rather than a limited workspace (e.g., tabletop). Our work will provide information about the effect of embodiments and gaze cues in room-scale interaction, which provides greater freedom of movement. Compared to this earlier work, our research makes the following novel contributions:

- Introduce eye-tracked gaze cues in an MR collaborative interface between both AR and VR conditions.
- Present the results of a formal user study that compares the effects of using different types of virtual gaze and viewpoint awareness cues in a room-scale collaborative MR interface.
- Discuss the benefits and the limitations of the current AR and VR platforms for supporting awareness cues in a remotely shared environment.
- Provide design guidelines for using virtual awareness cues in collaborative MR.

## Related Work

Our work combines and extends earlier research in MR collaboration, the remote embodiment in collaborative systems, and using visual cues for providing information about the head pose and eye gaze. In this section, we review earlier work in each of these areas and outline the research contribution we are making.

### Mixed-Reality Collaboration

MR collaborative systems combine AR and VR technologies to combine the strengths of each platform. Collaborative experiences in AR or VR are relatively common, but our research is concerned with interfaces that support collaboration between AR and VR views. One of the earliest was Kiyokawa's system (Kiyokawa et al., 1999) which allowed users to easily move between VR and AR views. The MagicBook interface (Billinghurst et al., 2001) allowed a user to fly inside a 3D scene and experience it from an ego-centric view in immersive VR, while a second user provided guidance from seeing the AR version of the scene from an exo-centric viewpoint. Similarly, Grasset et al. (2005) reported on a navigation task where one user looks down upon a virtual maze from an AR exocentric viewpoint, and help their partner, who is in a VR egocentric view, find their way out. They found that navigation assistance improved task performance but found no benefit of AR over VR for the exocentric view. The Vishnu interface (Le Chénéchal et al., 2016) supports collaboration between an expert in a VR display and a local worker in a video see-through AR system, where the expert uses virtual gestures to help the AR user complete a real-world task. Oda et al. (2015) developed a system in which an expert user in VR could use pointing and virtual object manipulation to help an AR user complete an object assembly task.

In these examples, both the AR and VR users were using head-mounted displays (HMDs). However, there are other display configurations that also support MR collaboration. For example, Stafford et al. (2008) used a tabletop display to provide an exo-centric view for collaboration with an AR user in an outdoor setting. The tabletop user could add virtual cues to guide the AR's user navigation. Sun et al. (2016) developed a system where a remote expert using desktop VR could provide virtual cues to a second user in an AR display. Tait (Tait and Billinghurst, 2015) developed a similar system where a desktop user placed 3D copies of real objects in a remote user's AR view to help complete an object placement task.

Several previous systems use different viewpoints in AR or VR to support different collaborative roles, such as a remote expert supervising another user who is performing a real-world task. In contrast, we present a system aimed at supporting AR and VR collaboration from a shared perspective. Previous systems showed the importance of awareness cues, such as virtual pointers (Greenberg et al., 1996; Duval et al., 2014; Oda et al., 2015) or hand gestures (Sodhi et al., 2013), to support effective communication. We explore using virtual cues to provide additional communication information, such as where a collaborator is looking using eye-gaze cues.

### Representing Head Pose and Eye Gaze

In face to face collaboration head pose and eye gaze are important communication cues, especially for the focus of attention. Traditional video conferencing systems have limited capability to portray gaze information due to a displacement of the camera viewpoint from a person's image and lack of support for spatial cues. However, when collaborating on a physical task, it is more important to provide awareness of where the user is looking rather than provide convincing face-to-face eye contact (Kuzuoka et al., 1999; Fussell et al., 2003; Lee et al., 2011). Visual cues representing view direction (Anthes and Volkert, 2005) can provide an observer with awareness of their collaborator's attention while allowing them to also view the same objects.

In collaborative AR and VR a virtual view frustum (Hindmarsh et al., 2000; Mogilev et al., 2002; Anthes and Volkert, 2005; Duval et al., 2014; Tait and Billinghurst, 2015; Gao et al., 2017; Muller et al., 2017) can be used to provide awareness of a user's head pose and field of view. Le Chénéchal et al. (2015) found trade-offs between the use of a virtual frustum and hand embodiments for providing remote navigation assistance. These AR and VR applications showed the benefits of using a virtual view frustum to show the user's focus of attention in a collaborative application, however, none of these works compared different types of cues in a formal user study.

Gaze can also be shared in collaborative applications to reveal more explicitly what a user is looking at. Several AR and VR systems have used gaze cues to help users communicate their intentions and provide an indicator for deictic references (Vertegaal, 1999; Steptoe et al., 2009; Gupta et al., 2016; Higuchi et al., 2016). Studies have shown that gaze cues can increase collaborators' sense of co-presence (Gupta et al., 2016) and are implicit pointers to facilitate communication (Gupta et al., 2016; Higuchi et al., 2016). However, most prior implementations share gaze in only one direction (e.g., from the local to remote user), whereas our system shares gaze cues mutually between both collaborators in a shared space. We also compare head pointing, view frustum, and eye gaze as awareness cues, in one of the first studies to incorporate virtual gaze cues in an MR space between AR and VR viewpoints.

### Remote Embodiment

Embodiment cues such as body position and gesture can also be important in remote collaboration. Embodiments are virtual representations that provide awareness (Gutwin and Greenberg, 1996) of a collaborator's activities by representing physical states, such as location, pose, movement or hand gestures. An early example is Telepointer (Greenberg et al., 1996), which replicates the motions of a remote cursor in a shared desktop workspace. Several techniques have been developed for sharing information about the state of the users' limbs such as arms (Tang et al., 2007, 2010; Doucette et al., 2013), hands (Tecchia et al., 2012; Sodhi et al., 2013; Wong, 2015), feet (Alizadeh et al., 2016), full-body avatar (Steptoe et al., 2008, 2012) in various remote collaboration platforms.

In order to convey gesture over a distance in a collaborative application, Tang et al. (2007) capture live images of arms working above a touch surface and rendered these arms on remote shared tabletop display. One limitation is that the captured hands or arms are 2D, and so appear flat, without any depth information. Several systems have captured users' hands in 3D, to provide information about depth and spatial relationships, and share hand embodiments through mobile AR (Sodhi et al., 2013), or a HMD in AR (Wong, 2015) or VR (Tecchia et al., 2012; Amores et al., 2015). Virtual embodiments have also been applied in collaborative MR systems using tabletop displays combined with AR (Stafford et al., 2008) or VR (Stafford et al., 2006). Oda et al. (2015) studied collaboration in MR systems, with AR and VR, but focused on virtual pointers and object replicas. In cases where objects cannot easily be indicated by hand gestures, researchers have explored alternate object referencing techniques such as raycasting (Duval et al., 2014), or virtual reconstruction of a selected scene region (Oda and Feiner, 2012). Finally, recent work on telepresence has demonstrated lifelike full-body reconstructions of distant persons, placed in a local environment (Beck et al., 2013; Maimone et al., 2013; Fuchs et al., 2014; Orts-Escolano et al., 2016).

This research shows that adding a representation of the user's body or gestures can improve collaboration in shared AR and VR experiences. They increase social presence, enable people to use natural non-verbal communication cues, and support shared interaction with the virtual content in the space. Our research builds on this work by applying such cues within a room-sized space, rather than a limited workspace. We also provide information about the effects of embodiments and gaze cues in room-scale interaction, which provides a greater freedom of movement.

From the previous work, we can see a number of researchers have explored collaboration between AR and VR spaces, but there have been few studies of the effect of virtual awareness cues, and none focusing on representing gaze between AR and VR users in Mixed Reality collaborative interfaces. The focus of our research is on the benefits of adding additional cues to an MR collaboration, providing information about the collaborator's focus of attention, such as a head pointer or view frustum to indicate where they are looking.

## User Study

We conducted a user study with 32 participants (16 pairs) to identify the effects of different combinations of awareness cues on the remote collaboration in MR. We were interested in view frustum, head-gaze, and eye-gaze cues, provided in addition to a baseline avatar and hand models.

### System Setup

**Figure 1:**
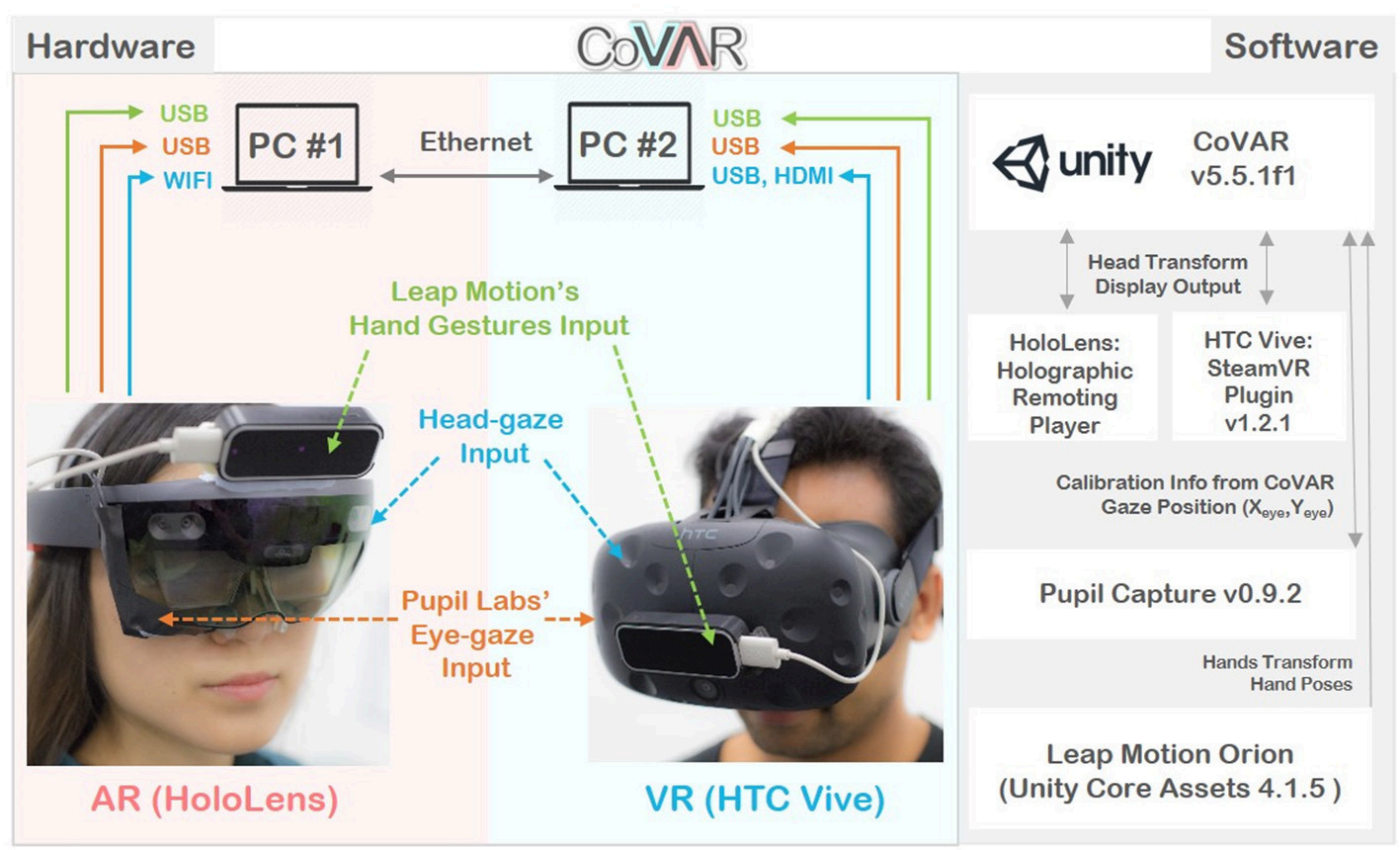
CoVAR's System overview.

To support remote collaboration between AR and VR users, we created CoVAR (Collaborative Virtual and Augmented Reality system), a multi-user collaborative system for MR with a client-server architecture. It was developed using Unity 5.5.1f1. For the AR side, we used the Microsoft HoloLens with Windows MR platform, and for VR, we used the HTC Vive display with SteamVR platform. CoVAR ran on a Windows 10 machine for both AR and VR sides. On the AR side, HoloLens was connected to CoVAR using the Holographic Remoting Player through a WIFI connection. The Vive was connected directly to the second machine through its compositor. The physical task space on the AR side was captured and reconstructed using the HoloLens into a 3D virtual model, then shared with the VR side. The two machines were connected by Ethernet with a TCP/IP connection and Unity Networking was used for data synchronization. The hardware and software overview of CoVAR is illustrated in Figure 1.

We designed the system in a way that either side, AR or VR user, can be the host server. With this design, we intend to support different use case scenarios that may arise. For example, a collaboration between a local worker in AR that hosts a session for remote assistance from the supervisor in VR. As the session ended or interrupted unexpectedly, the AR host would not lose the data following the termination of the session. Another scenario is when the local VR supervisor hosts a session for multiple remote AR workers to gather data for the supervisor's side to collect, assemble, and analyses. Figure 3 illustrates our use case in the study.

Our data such as the user's position and gaze's position were sampled every second. This was much lower than the hardware sensors' sampling rate such as HTC's Vive tracker at 90 Hz, Leap Motion (Leap_Motion Leap Motion Hand Tracking, 2018) at 120 Hz, Pupil Labs' eye tracker (Pupil_Labs Pupil Labs' Eye Tracker, 2018) at 200 Hz. The last immediate reading from each measurement along with the synchronized timestamp was recorded on both the AR and VR machine. The two machines' timer were synchronized at the start of the simulation.

#### System Interaction

To create a seamless collaborative experience, CoVAR provides common inputs across different platforms. The three fundamental inputs shared between AR and VR users were the head-gaze, eye-gaze, and hand gestures.

##### Head-gaze input

Head-gaze is input from the user's head movement. This data is provided by the Head Mounted Display (HMD)'s tracking data. For the HoloLens, the localization is provided by its integrated spatial mapping technology and for the HTC Vive, by the Lighthouse tracking system. The head-gaze location is taken as the point of intersection between a ray cast from the head's center toward the center of user's Field-of-View (FoV) and the first object it hits. To the users, their own head-gaze is represented by a blue-dot reticle in an inactive state and a blue-circle in an active state, as shown in Figures 4A,B, respectively. Note that the head reticle is the only visual cue that the users can see from the head-gaze cue.

##### Eye-gaze input

**Figure 2:**
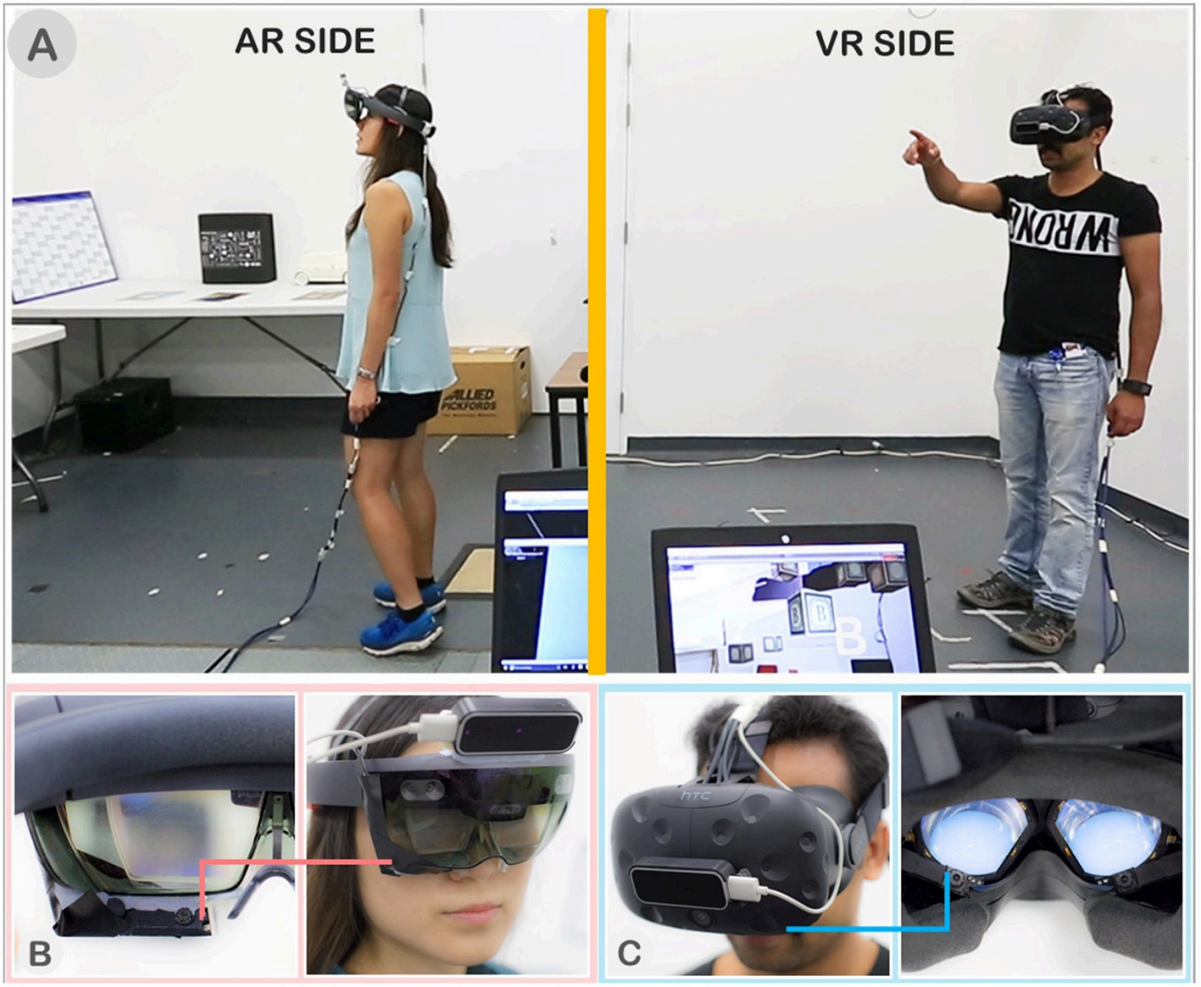
**(A)** Experimental space with AR on left and the VR user on right. **(B)** AR user wearing Microsoft HoloLens with single Pupil Labs camera for eye tracking. **(C)** VR user wearing HTC Vive (left) with dual Pupil Labs cameras.

To track user's eye-gaze, we mounted the Pupil Labs eye tracking (Pupil_Labs) into the HoloLens and Vive as shown in Figures 2B,C, respectively. We used the Pupil Labs Capture software for calibration and tracking. The eye-gaze location is taken as the point of intersection between a ray cast from the head's center position in the direction of the projected eye-gaze point and the first object it hits. The eye-gaze's location is represented to the user by an eye-shaped reticle as shown in Figure 4C. The eye reticle is the only visual cue that the users can see from the eye-gaze cue. To the users, the eye reticle is updated with the latest eye gaze position every frame. To the collaborator, a small moving average filter (*n* = 5) is applied to the eye gaze position to smooth out the gaze ray.

**Figure 3:**
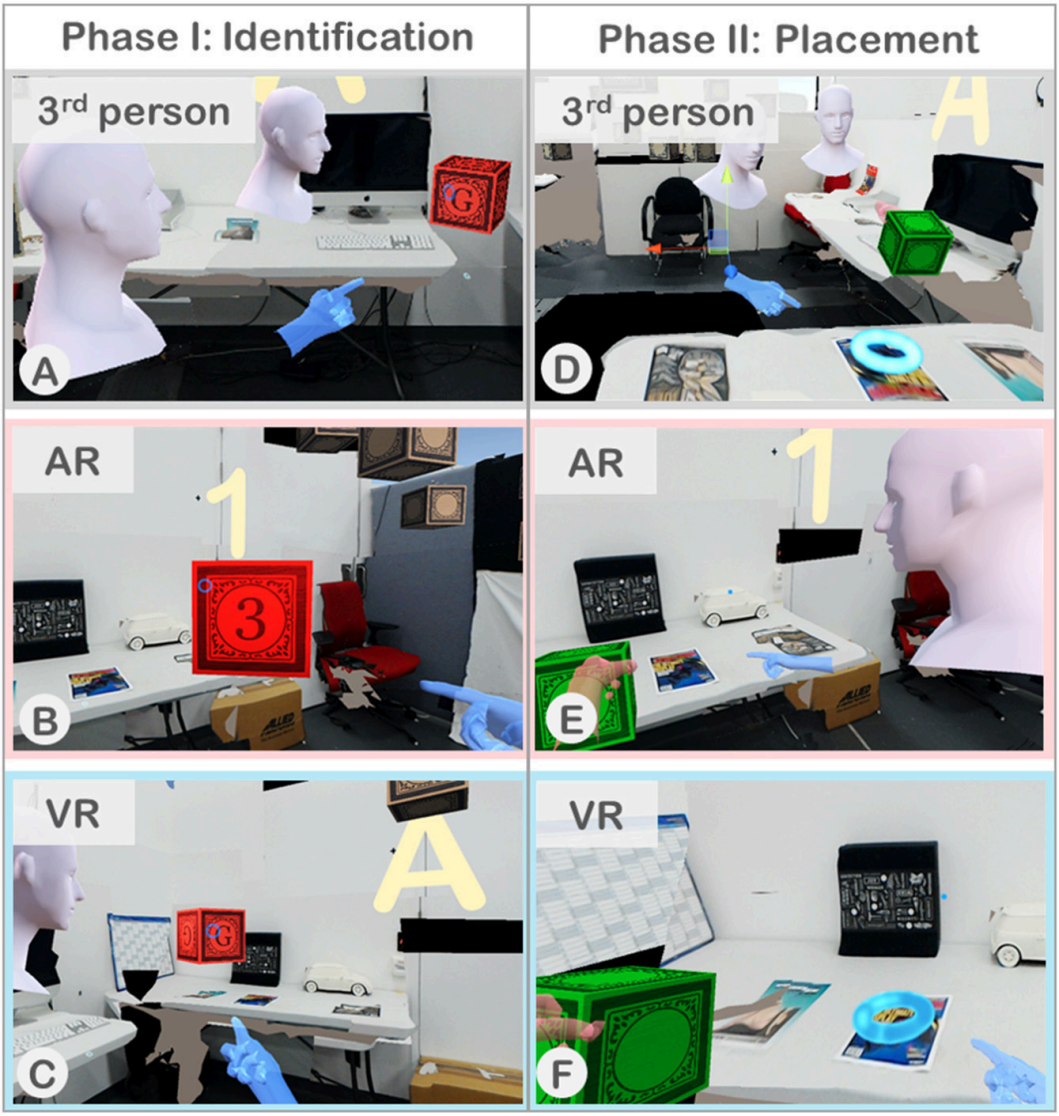
Collaborative search task: **(A)** AR and VR users search for the block with the correct letter (visible only to VR user) and number (visible only to AR user). **(B)** Users identify the correct block and move it to the placement target (blue ring). **(C)** AR user's search view—red indicates an incorrect block selection. **(D)** AR user grasps correct block and follows VR user's instructions to placement target (not visible to AR user). **(E)** VR user's search view. **(F)** VR user guides AR user to placement target.

**Figure 4:**
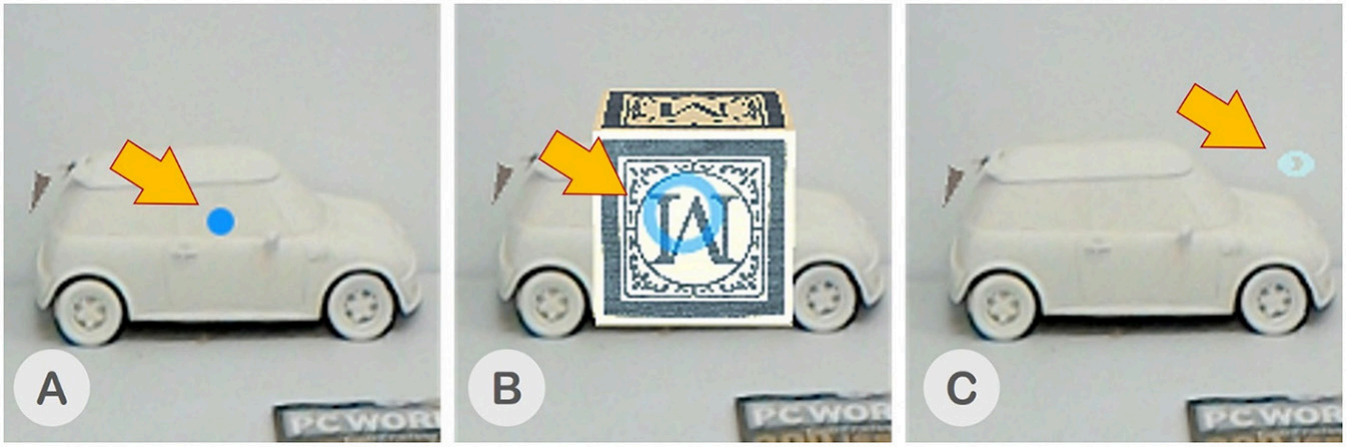
**(A)** Blue dot - an inactive head-gaze reticle, **(B)** Blue circle an active head-gaze reticle, and **(C)** Light blue eye image - an eye-gaze reticle.

##### Hand gestures input

The HoloLens supports only a small set of free-hand gestures as inputs, while the Vive relies on its controllers. We wanted to provide a common input method that encourages natural interaction across different platforms and so we integrated the Leap Motion (Leap_Motion) into the system for hand pose and gesture recognition. The Leap Motion sensors were mounted on top of the HoloLens and in front of the Vive as shown in Figure 1. While there are a number of gesture interactions implemented in CoVAR (e.g., pointing with ray, sweeping here/away, etc.), we only used the object grasping gesture in the user study.

#### Awareness Cues

To enhance the remote collaboration experience, CoVAR provides four visual cues to improve the users' communication; an avatar's head, avatar's hands, a Field of View (FoV) frustum cue, and a gaze cue. The avatar's head and hands are common cues.

##### Common cues

**Figure 5:**
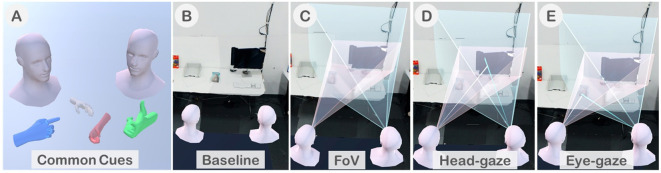
**(A)** Common cues including avatar's busts and hands, **(B)** Baseline condition in the user study, **(C)** FoV condition where AR frustum is in pink and VR in blue, **(D)** Head-gaze condition with FoV and head-gaze ray (co-gaze at the iMac), and **(E)** Eye-gaze condition with FoV and eye-gaze ray (co-gaze at the magazine).

These comprise of the avatar's head which represents the remote user's head to indicate the user's position and face direction, and the avatar's hands representing the user's hands. Although the local users can see their own hands animated with full degree-of-freedom (DoF) of control, in order to save the amount of data exchanged between the users, the remote collaborator's hands were represented with one of the four possible pre-defined hand poses. When the user's hand pose is recognized as one of the predefined poses, the hand is highlighted in different colors to indicate that a certain pose is visible to the remote user. The colors for the poses are neutral pose in gray, pointing in blue, grasping in red, and thumbs up in green. These common cues are shown in Figure 5A.

##### Field-of-view cue (FoV)

**Figure 6:**
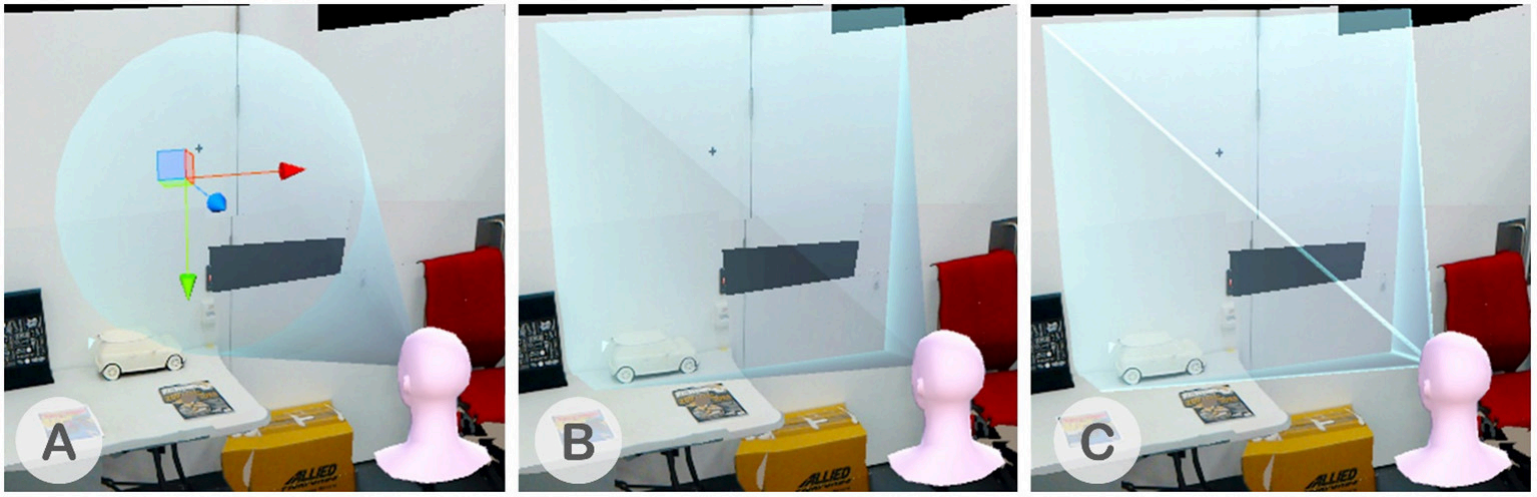
**(A)** Cone-shaped FoV, **(B)** Pyramid-shaped FoV, and **(C)** Highlighted-edge pyramid-shaped FoV.

The FoV frustum cue represents the view volume of the remote collaborator. Different display technologies support different sizes of FoV. We believe that it is crucial for the collaborators to be aware of each other's FoV limitation. The remote collaborator's FoV is represented by a frustum as shown in Figure 5C. While the smaller AR frustum in pink matches the size of the HoloLens's FoV (35°), the blue VR frustum has been reduced to half the size of the actual FoV as we found from pilot tests that it is difficult to recognize when it is in the actual size of the HTC Vive's FoV (110°). We also provide a spotlight that matched the shape of the frustum so that the user is aware of the area that the frustum intersects with the working surface. We designed several FoV representations for VR as shown in Figures 6A–C. We had brief sessions of user tests and found that the pyramid-shaped FoV with highlighted-edge work best for the collaborator.

##### Gaze cue

The gaze cue is shown as a ray representing the user's gaze direction. In case of the head gaze, it is a line emitting from the center of the user's head toward the center of the FoV frustum, up to the object being looked at which the gaze line intersects with (Figure 5D). For the eye-gaze, it is a line projected from the center of the user's head in the direction of the eye-gaze point in the FoV, up to the gazed object (Figure 5E).

#### Equipment

The hardware equipment used in this study was as follows: **VR Side:** (A) a Windows 10 laptop computer with an Intel Core i7-6700HQ at 2.6 GHz, 16 GB RAM, and NVIDIA GeForce GTX 1070, (B) HTC Vive Kit, (C) a pair of Pupil Labs eye trackers with a binocular mount for the HTC Vive HMD running at 120 Hz, (D) a Leap Motion sensor and VR mount unit; **AR Side:** (A) a Windows 10 laptop PC with an Intel Core i7-4800MQ at 2.7 GHz, 32 GB RAM, and NVIDIA GeForce GTX 780M, (B) Microsoft HoloLens, (C) a Pupil Labs eye tracker, and (D) a Leap Motion sensor on a custom-made mount unit. Both computers were networked through Ethernet connection. Videos were recorded in each trial using a DSLR camera so that the whole experimental space and all verbal communication could be captured in a single video. Data generated from the users' movement and interaction was also recorded for each trial.

#### Experimental Space

**Figure 7:**
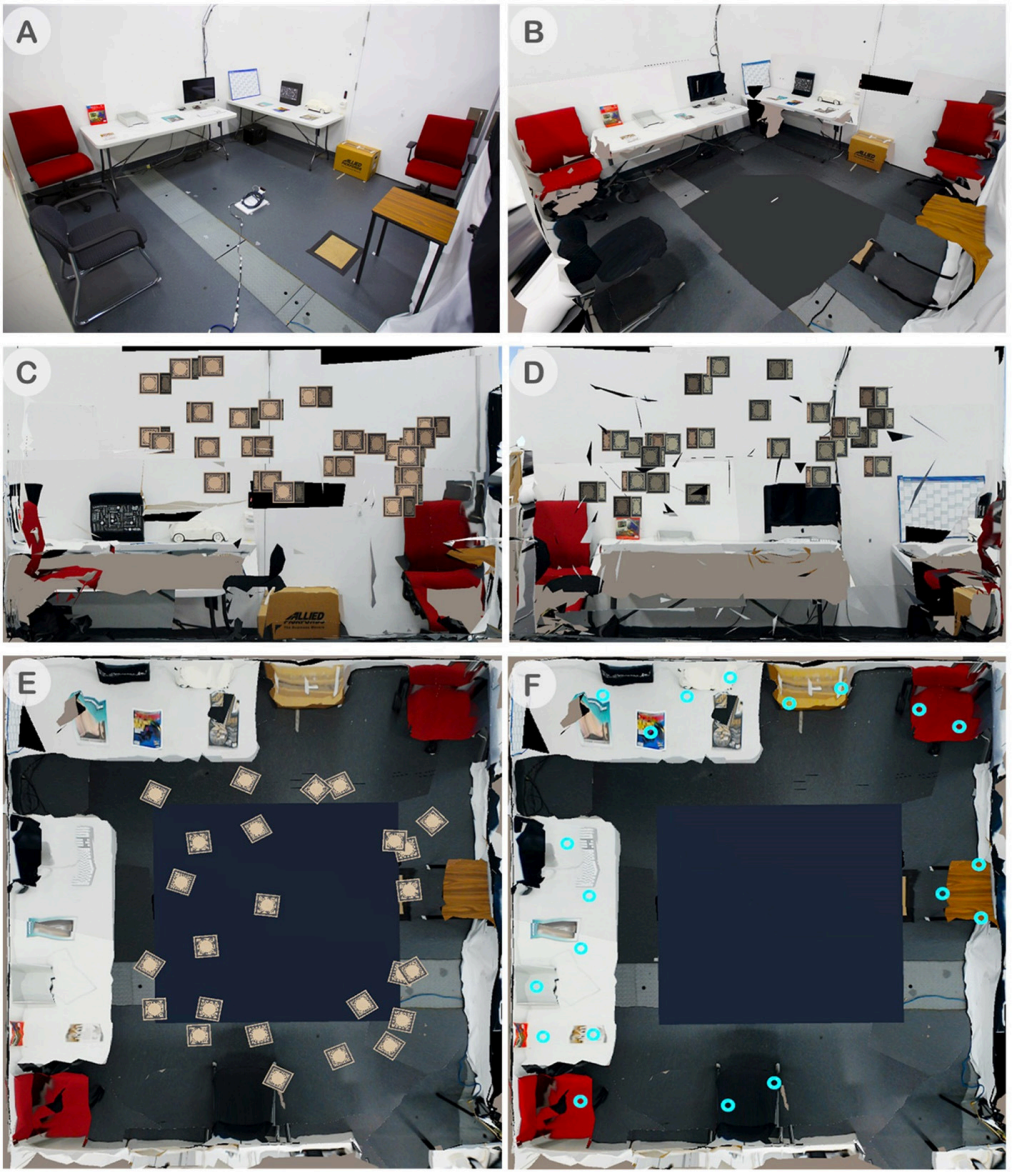
**(A)** AR space with real furniture and props. HoloLens was placed on the ground in the center of the room at the beginning of the simulation to establish the room origin to align to the VR space, **(B)** The reconstructed room for VR user using the HoloLens's spatial map with image textures, **(C)** The top view of the shared space showing an example of 25 randomized block spawn locations, **(D)** Showing all the 20 possible spawn locations of the placement target (blue rings), **(E)** Front wall of the workspace view, and **(F)** Left wall of the workspace view.

We conducted our study in a lab space with 5 m tall ceiling. The experimental space was divided with 1.8 m tall partitions into two sides for AR and VR spaces such that users could not see each other but could still talk to each other, as shown in Figure 2A. This was similar to experimental set-ups used in prior work (Gupta et al., 2016). Each side occupied an area of 3.5 by 3.5 meters. The AR side was furnished with furniture and props for spatial references, while the VR side was left empty. For scene reconstruction, we used the HoloLens Image-based Texturing software to create the spatial map and captured texture images. The original AR space is shown in Figure 7A and the result of the reconstruction for the VR side is shown in Figures 7B.

### Study Design and Variables

We designed the experiment to be within-subjects where we investigated the effects of three combinations of awareness cues, the only independent variable, and one baseline condition where no additional awareness cue was present. There were eight different dependent variables including both objective and subjective. We did not conduct a factorial design because the combinations of all the awareness cues that we would like to examine would yield too many conditions, therefore, we only selected those we felt the most compelling for this study. We had considered a factorial design where each visual cue was treated as an individual independent variable. However, this would result in 16 conditions (four factors and each factor with two levels). As an alternative, we chose the most interesting conditions for the purpose of our user study.

#### Awareness Cues

Awareness Cues *(independent variable, within-subject)*: There were four different levels of awareness cue variable. The three cues (except for the Baseline) were identified after a pilot-study with interaction designers in our group. We counter-balanced the order of the awareness cues using a 4 × 4 Balanced Latin- square. For gaze-based interaction, head-gaze input was used in all conditions as a control variable. The awareness cue conditions were:

- *Baseline*: In the Baseline condition, we showed only the head and hands of the collaborator in the scene. The head and hands were presented in all conditions (Figure 5B).
- *Field-of-view (FoV)*: We showed the FoV frustum of each collaborator to the other. This enabled collaborators to understand roughly where their partner was looking and how much area the other person could see at any point in time. All the other conditions, except the Baseline, had the FoV cue in them (Figure 5C).
- *Head-gaze (FoV* + *Head-gaze ray)*: Together with the FoV frustum, we also showed a ray originating from the user's head to identify the center of the FoV, which provided a more precise indication where the other collaborator was looking at (Figure 5D).
- *Eye-gaze (FoV* + *Eye-gaze ray)*: In this cue, we showed a ray originating from the user's eye to show exactly where the user was looking at. It provided a more accurate identification of the collaborators gaze directions (Figure 5E).

#### Dependent Variables

We had eight objective and subjective dependent variables, as shown in Table 1. For the objective variables, we measured the rate of mutual gaze, the total task completion time, the number of hand gestures performed, the distance traveled, the distance difference between the two collaborators at a given time. The subjective variables were subjective feedback on the usability of the system (Brooke, 1996), social presence questionnaire (Harms and Biocca, 2004), and semi-structured interviews. We also video recorded participants to analyze their behavior.

**Table 1:** Measurements and key results of this experiment.

**Measure type**	**Variable name**	**Key results**
Performance metrics	Rate of mutual gaze (objects identified/minute) Task completion time (seconds)	Head-gaze and Eye-gaze had more rate of mutual gaze than Baseline No significant difference
Observed behavior	Number of hand gestures Physical movement (meters) Distance between collaborators (meters)	Head-gaze and Eye-gaze needed less hand pointing than Baseline Head-gaze required least physical movement in the scene Eye-gaze condition had collaborators in closest proximity and Baseline had them most dispersed
Subjective surveys	Usability Social presence Semi-structured interview	Head-gaze was most easy to use and useful Baseline and FoV were more confusing than Head-gaze Baseline had least co-presence, others were similar FoV had worst attention allocation ratings and Eye-gaze was best Head-gaze had best perceived message understanding and perceived behavioral independence, baseline was worst in both Head-gaze preferred mostly AR users reported higher difficulty than VR users

### Hypotheses

We postulated the following hypotheses.

- H1: The Baseline condition does not provide any additional cue, so we hypothesized that it would be the worst condition in terms of all performance metrics and behavioral observation variables (Table 1).
- H2: The Head-gaze and Eye-gaze conditions provide a gaze pointer to identify the center of the FoV frustum and exact eye-gaze location respectively, which will enable users to perform better using these cues than the FoV only condition.
- H3: In terms of subjective opinions, the Head-gaze and Eye-gaze will be favored more than the Baseline condition, as not having a cue will increase the collaborators' task load.
- H4: The Baseline condition requires more physical movement from the collaborators as they need to move around and look at their collaborator's avatar.
- H5: The Baseline condition requires a larger distance separating the collaborators so that they could see each other's avatar.

### Task and Procedure

To promote collaboration and to study the effect of awareness cues on collaboration, we designed an experimental task which involved search and manipulation of virtual objects, called “Gaze and Place.” This task had two phases, search and placement, where both phases required active collaboration while each phase involved different roles between collaborators. In Gaze and Place, participants had to collaboratively find a virtual block located in the scene and to place them at a target location in relation to the physical objects. All the virtual objects were placed relative to the physical scene on the AR side, while for the VR user, a virtual reconstruction of the physical space on AR side was shown as a spatial reference together with the virtual objects. At the beginning of the task, 25 virtual blocks were randomly placed in the scene within the designated area surrounding the participants standing at the center of the scene. Each virtual block had a number and a letter on it, but the AR user could only see the number and the VR user could only see the letter. The blocks were initially shown as blank with a hidden number/letter, and only when each user gazed at it using the head reticle, would the number or letter be revealed.

**Figure 8:**
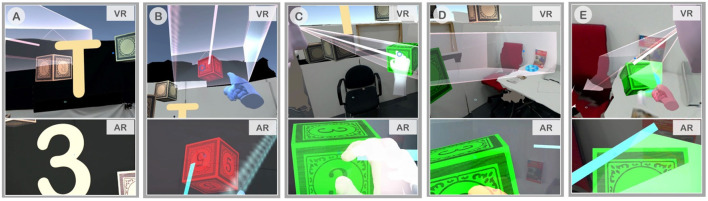
Snapshots from the actual footage captured during the collaboration, (top row) VR user's cropped screen captures, and (bottom row) AR user's full view captured by HoloLens, **(A)** Both users needed to find a 3T block, **(B)** VR user pointed at the block, while both users co-gazed at the incorrect block, 5T, **(C)** 3T was found and the AR user was grabbing it, **(D)** VR user found the placement target in front of the red book, and **(E)** AR user placed the block at the target and the trial was completed.

In the first phase of searching and identification (Figures 3A,C,E), the users collaborated to find a block with a correct combination of a number and a letter (e.g., 1A, 5D, 3C). The number/letter that the users needed to find collaboratively was displayed on the four walls of the room (Figure 8A). Among the 25 blocks randomly spawned, there were multiple blocks with the same number on the AR side and the same letter on the VR side but only one with the correct combination. The users had to use verbal communication and visual awareness cues provided in each condition to identify the correct block. Each user could gaze at the block to reveal the hidden number or letter and then examine the block individually. When both users looked at the same block together (mutual gaze), the block's color slowly changed its color to indicate if it is the correct block they are looking for. If the block turned red, it was an incorrect one (Figure 8B), and if it turned green, it was the correct block (Figure 8C). In this phase of the task, each user has the same role and so it is a symmetric collaboration task.

In comparison, the second phase of the task, placement (Figures 3B,D,F), is an asymmetric collaboration as the users have different roles—instructor and worker. After the correct block was identified, one of the users could move this green block using the grasping gesture by making a fist pose within the block. This user is called a worker (Figure 8C). Once the worker touched the green block, the roles between the users were fixed and could not be changed. Only the other user, who did not touch the block, would be able to see the placement target location represented by a glowing blue ring where the worker had to place the block at. We call this user the instructor. The participants were told that the first person who touched the block would be moving it, therefore they had to reach an agreement on their roles prior to someone touching the block. The placement target was randomly chosen from one of the twenty possible spawn points (Figure 7D). The instructor had to locate the placement target and direct the worker to place the green block at the target location (Figure 8D). Once the worker placed the block at the placement target, after a second, a semi-transparent green cube would appear to both users to confirm the successful placement and to indicate the trial is completed (Figure 8E).

At the beginning of each study, we explained the experimental procedure verbally and provided a demonstration where two experimenters practiced the task. Users also trained themselves with the system by performing a practice trial of the task in each condition before the data collection began. We asked participants to fill out a demographic form. After each condition, participants were asked to fill out a survey that included social presence (Harms and Biocca, 2004), the system usability scale (Brooke, 1996), and general usability questions. At the beginning of each condition, we asked and reminded the participants to communicate verbally and to use the awareness cues as they preferred. They were asked to finish the task as quickly as possible. In the second phase, we gave them the freedom to decide who would take the role of the worker and the instructor, as we wanted to find out the subjective preference in performing the task.

Overall, there were four conditions in an experimental session and for each condition, participants had to perform eight trials. The entire experiment took approximately 1.5 h to complete on average per pair of participants.

### Participants

We recruited 16 pairs, 32 participants in total from the general population using online advertisement, email contacts, and meetup groups. Except for two pairs, all the other pairs knew each other socially and had at least one interaction before the experiment. Those who didn't have a prior interaction were asked to introduce themselves to each other and carry a conversation for about 5 min to break the communication barrier. Out of the 32 participants, 9 were females. Five pairs had mixed-gender, two pairs were females only, and nine males only. The age range of the participants was between 20 and 55 years with a mean of 30.8 years (*SD* = 7.7). Six participants did not have any prior experience with VR and 10 participants had no prior experience with AR. Seven participants had no prior experience with any of the HMDs. This study had been approved by the University of South Australia's Human Research Ethics Committee.

### Results

In the following, we first present the analysis of the objective data, followed by the subjective data. Overall, in this experiment we collected 4 (conditions) × 8 (trials per condition) × 16 pairs = 512 data points for objective variables. For subjective variables, we collected 4 (conditions) × 32 (participants) = 128 data points. All data was prepared and analyzed using IBM SPSS^TM^ version 21. We used one-way repeated measured ANOVAs (α = 0.05) for all the variables and followed by pair-wise comparisons with the Bonferroni correction for the results with a significant difference. Data were checked for normality and sphericity, and no deviation from the assumptions was found.

#### Objective Data

##### The rate of mutual gaze

**Figure 9:**
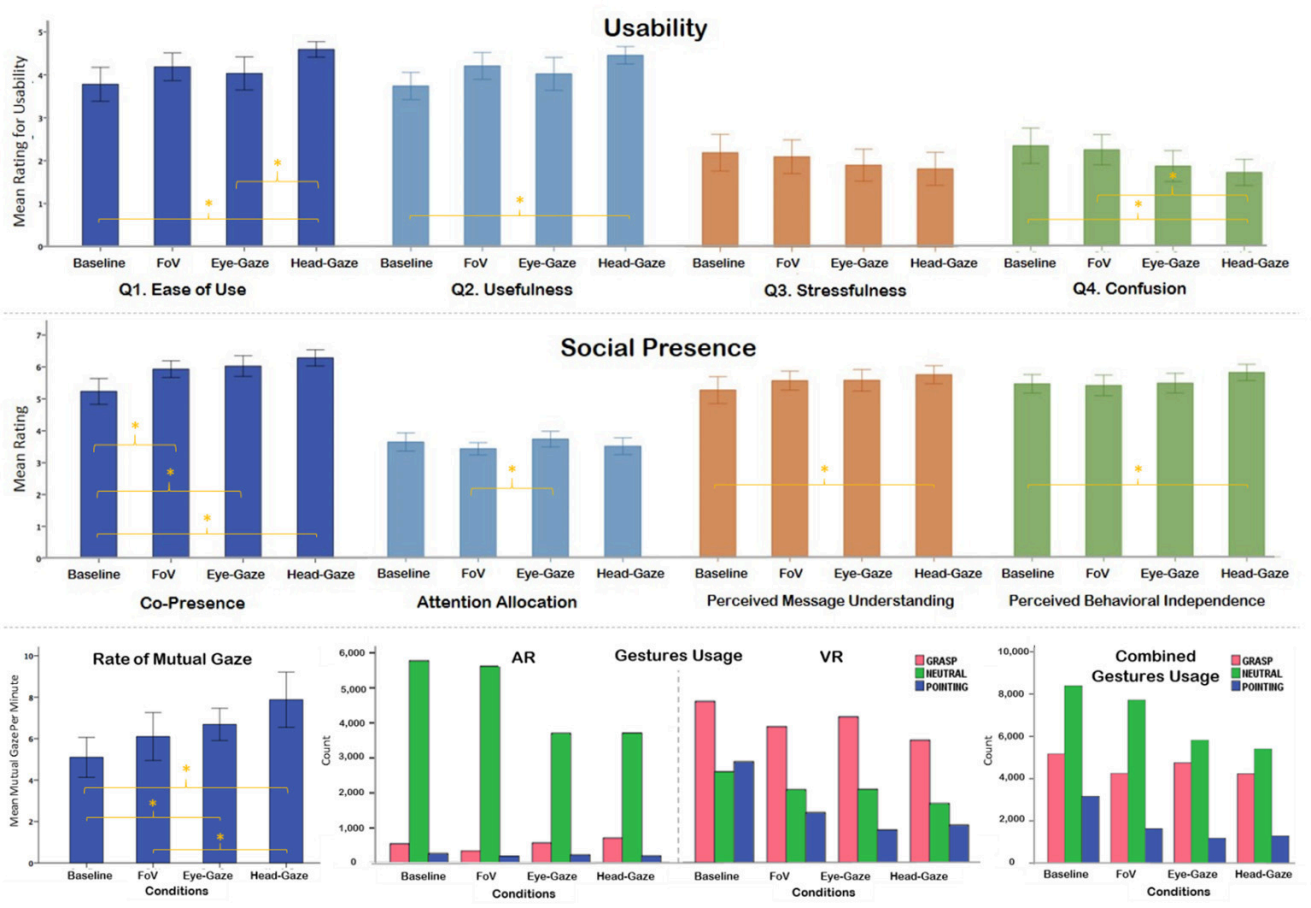
Plots (whiskers represent ±95% confidence interval)—Usability Ratings **(Top)**, Social presence rating for four different sub-scales **(Middle)**, Rate of mutual gaze **(Bottom Left)**, AR and VR gestures usage **(Bottom Middle)**, and combined gestures usage of both AR and VR **(Bottom Right)**.

We counted how many times collaborators looked at the same block during the identification task, which enabled them to identify whether it was the correct block. The number of mutual gazes was counted for the entire identification period and the rate of mutual gaze was calculated by dividing the total mutual gaze count by the identification task completion time (in minute). We noticed a significant difference *F*_(3, 45)_ = 7.94, *p* < 0.001, $\eta_{\text{p}}^{2}$ = 0.35 (Table 2). *Post-hoc* analysis showed that the Baseline condition had a significantly fewer number of mutual gaze targets per minute than the Eye-gaze and the Head-gaze conditions. The FoV also had significantly less mutual gaze than Head-Gaze condition (Figure 9).

**Table 2:** Mean (standard deviation) values of objective variables for mutual gaze, task completion time, and hand gestures.

**Conditions**	**Mutual gaze (count/min.)**	**Task completion time (sec.)**	**Hand gestures (count per sec.)**		
			**Pointing hand pose**	**Grasp hand pose**	**Neutral hand pose**
Baseline	5.1 (1.8)	79.2 (31.2)	3,159	5,164	8,383
FoV	6.1 (2.2)	69.1 (23.0)	1,632	4,237	7,711
Eye-gaze	6.7 (1.5)	79.15 (38.3)	1,176	4,746	5,806
Head-gaze	7.9 (2.5)	65.2 (19.5)	1,281	4,220	5,401

##### Total task completion time

We calculated the time taken by participants from the beginning of a trial until the block was successfully placed at the target location, the combined task completion time for the identification task and the placement task. While we found Head-gaze to be the fastest condition and Baseline to be the slowest (Table 2), the differences were not significant (*p* = 0.15). We calculated the time taken by participants from the beginning of a trial until the target block was successfully found. While we found Head-gaze to be the fastest condition and Eye-gaze to be the slowest, the differences were not significant (*p* = 0.1). We did not find a significant difference for *time to place* (*p* = 0.44), where Baseline was slowest, and others were similar.

##### Gestures usage

There were three different gestures (or hand postures) that participants could use: pointing, grasp, and neutral. We recorded the gesture used every second for the entire duration of the trial, hence the number counted for each gesture shown in Table 2. We calculated the usage ratio for each gesture by dividing the given gesture count with the total task completion time (Equation 1) and compared between the same type of gesture between conditions. We found that the number of gestures used in different awareness cues was varying significantly χ2_(6)_ = 880.82, *N* = 52,916, *p* < 0.001 (Figure 9). We particularly noticed that in the Head-gaze condition the number of hand gestures used was the lowest among all the conditions (Table 2). The number of pointing gestures used was highest in the Baseline condition and lowest in the Eye-gaze condition. The Head-gaze condition has a similar number of pointing gestures as the Eye-gaze condition. This finding makes sense as participants tried to use the ray available in Head-gaze and Eye-gaze conditions in place of the hand pointing in the Baseline and FoV conditions, which is an indication of later two conditions being physically demanding.

(1) $$\begin{array}{r} \text{Selected Gesture Usage Ratio}=\frac{\text{Selected gesture count per second}}{\text{Task completiontime}} \end{array}$$

##### Physical movement in the scene

We calculated the total movement (in meters) of participants in the environment as an indication of the physical load (Table 3). First, a sum of distance traveled by both collaborators together yielded a *p*-value very close to the significance level, *F*_(3, 45)_ = 2.54, *p* = 0.07, $\eta_{\text{p}}^{2}$ = 0.15. Further investigating the results, a pair-wise comparison showed a significant difference between Baseline and Head-gaze with p = 0.04. When we investigated differences for AR and VR collaborators separately, we didn't notice a significant difference for AR users but there was a significant difference for VR users F(3, 45) = 3.06, *p* = 0.04, $\eta_{\text{p}}^{2}$ = 0.17 (Figure 9). In VR, users moved significantly more in the Baseline condition than in the Head-gaze condition. Given that the XY plane was the ground (omitting the height difference on the z-axis), the Euclidean distances between the current AR user's position, P(x_i_, y_i_), and the previous user's position, P(x_i−1_, y_i−1_), were summed up every second for the duration of the trial. The same calculation was also performed for the VR user's current position, Q(x_i_, y_i_), and the previous position, Q(x_i−1_, y_i−1_). The addition of the two summed distances yielded the total movement for each trial (Equation 2).

(2) $$\begin{array}{rc} \text{Total Movement}= & \sum_{i=1}^{n}\sqrt{{\left(px_{i}-px_{i-1}\right)}^{2}+{\left(py_{i}-py_{i-1}\right)}^{2}}\\ \;+\sqrt{{\left(qx_{i}-qx_{i-1}\right)}^{2}+{\left(qy_{i}-qy_{i-1}\right)}^{2}} \end{array}$$

**Table 3:** Mean (standard deviation) values of objective variables for physical movement and distance between collaborators.

**Conditions**	**Physical movement (meters)**			**Average distance between collaborators (meters)**
	**AR**	**VR**	**Total (AR + VR)**
Baseline	67.6 (25.8)	62.7 (26.7)	130.3 (46.9)	0.73 (0.2)
FoV	59.6 (19.3)	53.5 (13.3)	113.1 (23.0)	0.68 (0.2)
Eye-gaze	59.4 (32.3)	49.7 (23.5)	109.1 (52.6)	0.64 (0.1)
Head-gaze	54.6 (18.5)	49.6 (14.9)	104.3 (26.8)	0.67 (0.2)

##### Average distance between collaborators

We measured the distances collaborators maintained between them to perform the task as a measure of behavioral differences (Table 3). The average distance between collaborators was calculated from the Euclidean distance between the AR user's position, P(x, y), and the VR user's position, Q(x, y) (Equation 3), given that the XY plane was the ground and the user positions were sampled every second and omitting the difference in heights. In the Eye-gaze condition collaborators maintained the closest proximity between them, while in Baseline, they were separated most. However, we did not find any significant difference between the conditions (*p* = 0.08).

(3) $$\begin{array}{rcl} \text{Avg Dist}. & = & \frac{1}{n}\sum_{i=1}^{n}\sqrt{{\left(px_{i}-qx_{i}\right)}^{2}+{\left(py_{i}-qy_{i}\right)}^{2}} \end{array}$$

#### Subjective Data

We collected three sets of subjective data. Usability and social presence surveys were answered after each condition and a semi-structured interview was conducted post session.

##### Usability

We asked four questions in this section. The questions were rephrased from the standard usability questionnaires (Brooke, 1996) for our purpose. (1) How easy was it to use the cue? (2) How useful was the cue for collaboration? (3) How stressful was it to use the cue? and (4) How confusing was the cue to understand? Participants answered the questions on a Likert-scale of 1-5 where 1 = Strongly Disagree and 5 = Strongly Agree (Table 4 and Figure 9).

**Table 4:** Mean (standard deviation) values of usability.

**Conditions**	**Usability (5-point Likert scale where 1 = Strongly Disagree and 5 = Strongly Agree)**			
	**Ease of use**	**Usefulness**	**Stressfulness**	**Confusion**
	***How easy was it to use the cue?***	***How useful was the cue for collaboration?***	***How stressful was it to use the cue?***	***How confusing was the cue to understand?***
Baseline	3.8 (1.1)	3.6 (0.9)	2.2 (1.2)	2.3 (1.2)
FoV	4.2 (0.9)	4.2 (0.9)	2.1 (1.1)	2.3 (1.0)
Eye-gaze	4.0 (1.0)	4.0 (1.0)	1.9 (1.0)	1.8 (1.1)
Head-gaze	4.6 (0.5)	4.2 (0.9)	1.8 (1.1)	1.7 (0.9)

For ease of use, we found a significant difference *F*_(3, 93)_ = 5.64, *p* = 0.001, $\eta_{\text{p}}^{2}$ = 0.15. Subjects felt that the Head-gaze condition was significantly easier to use than the Baseline and Eye-gaze conditions. For usefulness, we found a significant effect *F*_(3, 93)_ = 4.8, *p* = 0.004, $\eta_{\text{p}}^{2}$ = 0.13. The Head-gaze condition was significantly more useful than the Baseline condition. For stressfulness, we didn't find any significant effect and all conditions were rated similarly being not so stressful with means ranging between 1.8 (Head-gaze) and 2.2 (Baseline). For confusion to understanding, there was a significant effect of conditions *F*_(3, 93)_ = 5.8, *p* = 0.001, $\eta_{\text{p}}^{2}$ = 0.16, where the Head-gaze condition was significantly less confusing to use than the Baseline and FoV conditions.

##### Social presence

We administered a social presence questionnaire following Harms and Biocca (Harms and Biocca, 2004) with a 7-point Likert scale (1: Strongly Disagree ~ 7: Strongly Agree). However, to reduce the load on participants to finish the experiment and nature of the collaborative task we only included questions from the sub-scales of co-presence, attention allocation, perceived message understanding, and perceived behavioral independence. We noticed a significant effect of conditions in all sub-scales of social presence (Table 5 and Figure 8).

**Table 5:** Mean (standard deviation) values of social presence.

**Conditions**	**Social presence (7-point Likert scale where 1 = Strongly Disagree and 7 = Strongly Agree)**			
	**Co-presence**	**Attention allocation**	**Perceived message understanding**	**Perceived behavioral independence**
Baseline	5.2 (1.1)	3.7 (0.8)	3.7 (0.8)	5.7 (0.8)
FoV	5.9 (0.7)	3.5 (0.6)	3.5 (0.6)	5.6 (0.9)
Eye-gaze	6.0 (0.8)	3.8 (0.7)	3.9 (0.8)	5.7 (0.8)
Head-gaze	6.3 (0.7)	3.6 (0.8)	3.6 (0.8)	6.1 (0.7)

For co-presence, we found a strong effect *F*_(3, 93)_ = 12.96, *p* < 0.001, $\eta_{\text{p}}^{2}$ = 0.3. The Baseline condition was scored significantly lower than all other conditions Head-gaze (*M* = 6.3, *SD* = 0.70), Eye-gaze (*M* = 6, *SD* = 0.84), and FoV (*M* = 5.9, *SD* = 0.72). For attention allocation, we noticed a significant effect of *F*_(3, 93)_ = 2.8, *p* = 0.045, $\eta_{\text{p}}^{2}$ = 0.1. The FoV condition was rated significantly lower than the Eye-gaze condition. Perceived message understanding had a significant effect of condition as well *F*_(3, 92)_ = 3.85, *p* = 0.012, $\eta_{\text{p}}^{2}$ = 0.1. Here we noticed that Baseline was significantly lower than Head-gaze. Finally, for the perceived behavioral independence we found a significant effect of *F*_(3, 92)_ = 3.28, *p* = 0.024, $\eta_{\text{p}}^{2}$ = 0.1. The Head gaze condition was rated significantly higher than the Baseline condition.

##### Semi-structured interview

We administered a semi-structured interview with both collaborators together post-session. We primarily asked them about their general experience in terms of what they did and did not like and what strategies they used to perform the task. Almost unanimously all participants reported difficulties of performing the task using the Baseline condition and argued in favor of the FoV guidance. Out of the 16 pairs, in 12 pairs both collaborators had the same choice of the favorite cue. Among those 12, 10 pairs favored the Head-gaze cue and two pairs favored the Eye-gaze cue. In the rest of the four pairs, collaborators had different favorite cues. Four of the users favored Head-gaze, three favored Eye-gaze, and one favored the FoV only condition.

Three participants commented about the Eye-gaze condition being confusing. A couple of participants mentioned that the opacity of the FoV cue should be reduced; otherwise it makes looking through the FoV harder. Two participants asked for the FoV condition to be adaptive to the position of the collaborator. For example, when both of the collaborators were at the same location the FoV can be hidden and shown again when they move away from each other.

The majority of the participants mentioned the ray of the Head-gaze and Eye-gaze conditions being helpful in identifying the exact block which the other collaborator was looking at. One of the most common dislikes participants reported was the weight of the HMDs, particularly when worn for a long time. Most of the participants using the AR display complained about the smaller field of view of the display and expressed difficulties in following the VR collaborators movement. After the interview, several groups wanted to try out the other environment and those who did all commented that the task was much easier in the VR side.

## Discussion

Our study provides some objective and subjective evidence that support the benefits of having awareness cues in enhancing user collaboration. Table 1 provides a complete summary of the study results. Although, we could not find any significant difference in terms of task completion time to support our hypothesis, H1, to claim a performance benefit of providing the awareness cues; we found that Head-gaze and Eye-gaze had significantly higher rate of mutual gaze than the Baseline condition. We also found that those two conditions also required significantly fewer pointing gestures comparing to the Baseline. H2 was not accepted as the FoV condition was not significantly different to the Eye-gaze and Head-gaze conditions. Hypothesis H3 was accepted as the Baseline condition was scored significantly lower than the Head-gaze and Eye-gaze conditions in most of the subjective measures. In terms of the physical movement, the Head-gaze condition required significantly less movement than the Baseline condition supporting H4. We found that the Eye-gaze condition had the collaborators positioned significantly closer than the Baseline, partially fulfilling H5, which hypothesized that the Baseline condition would have the collaborators further apart than the other conditions.

Overall, we could not strongly claim the benefits of providing the awareness cues for MR collaboration from the results of this study, however, these preliminary findings did provide some evidence and insights into the nature of collaboration between the AR and VR users. In the rest of this section, we grouped the significant findings into common themes and discuss on how the results of one measurement (e.g., a performance metric) support (or contradict) the results of another (e.g., usability).

### The Breadth and Depth in Coordination

The introduction of awareness cues in the FoV, Eye-gaze and Head-gaze conditions helped to improve the awareness of the collaborators as the Baseline condition was found to be significantly worse than the other conditions in terms of co-presence. The FoV frustum indicated the view-direction and the limit of vision of the user. This information was crucial, especially for the AR users who had a small FoV so that their collaborator would be aware of their limited vision. The gaze ray indicated a precise location of where the user was looking and helped to disambiguate an object of interest from its neighbors.

#### Precision and Efficiency of Gaze

To compare the performance of each condition, the best indicator was the total number of blocks that users gazed together within the given period. We noticed that the Head-gaze and Eye-gaze conditions had a significantly higher rate of mutual gaze over the Baseline condition. In both the Head-gaze and Eye-gaze conditions, the total distance traveled by the VR users was also significantly lower than the Baseline condition. This meant that the gaze cue could help reduce the movement of the VR user. Moreover, the Baseline condition was found to have a significantly higher number of gestures used than both gaze conditions. these findings provided strong evidence to support our belief that the gaze cue is crucial for improving remote collaboration and reducing task load.

#### Head-Gaze Was Most Useful

We found that the Head-gaze condition was rated significantly higher than the Baseline and Eye-gaze conditions in term of ease of use and usefulness. It was also the least confusing to use and significantly better than the Baseline and FoV conditions. This was expected as Head-gaze offered both awareness cues in FoV and gaze cue and Head-gaze input was used as the default interaction method for revealing the character on the block, utilizing the implicit nature of shared interaction and awareness cue. During pilot tests, we found that if we used Eye-gaze as an input to reveal the block's character, the VR user could scan the blocks very quickly due to the incredible speed of eye movement and the larger FoV of the VR display. However, to prevent a confounding factor in the study, we used Head-gaze input for all conditions including the Eye-gaze condition.

### AR vs. VR Experience

Although both users had an equal role in the study (note that they were free to choose their roles in the second phase), the imbalance of power of the different platforms influenced the user's behavior and the effect of having different awareness cues significantly.

#### VR Dominance

By sharing the workspace reconstruction to the VR side, the VR user could understand and use the spatial information to better collaborate with the local AR user as if s/he was there. Furthermore, with a wide field-of-view VR display, the VR user also possessed a greater peripheral vision of the virtual workspace than the AR users. This means that the VR users could locate blocks, or the placement target faster than the AR users. We also noticed that the VR users mostly dominated the gesture interaction. Although, the same hand tracking technology was used for both AR and VR sides, the limited FOV of the HoloLens hindered the user experience using freehand gestures for pointing or grasping an object. This was because the visual cue was the only feedback that indicated the hand tracking status, outside the FOV, it was difficult to know if the hand tracking was still functioning.

#### Circumstantial Leader

**Figure 10:**
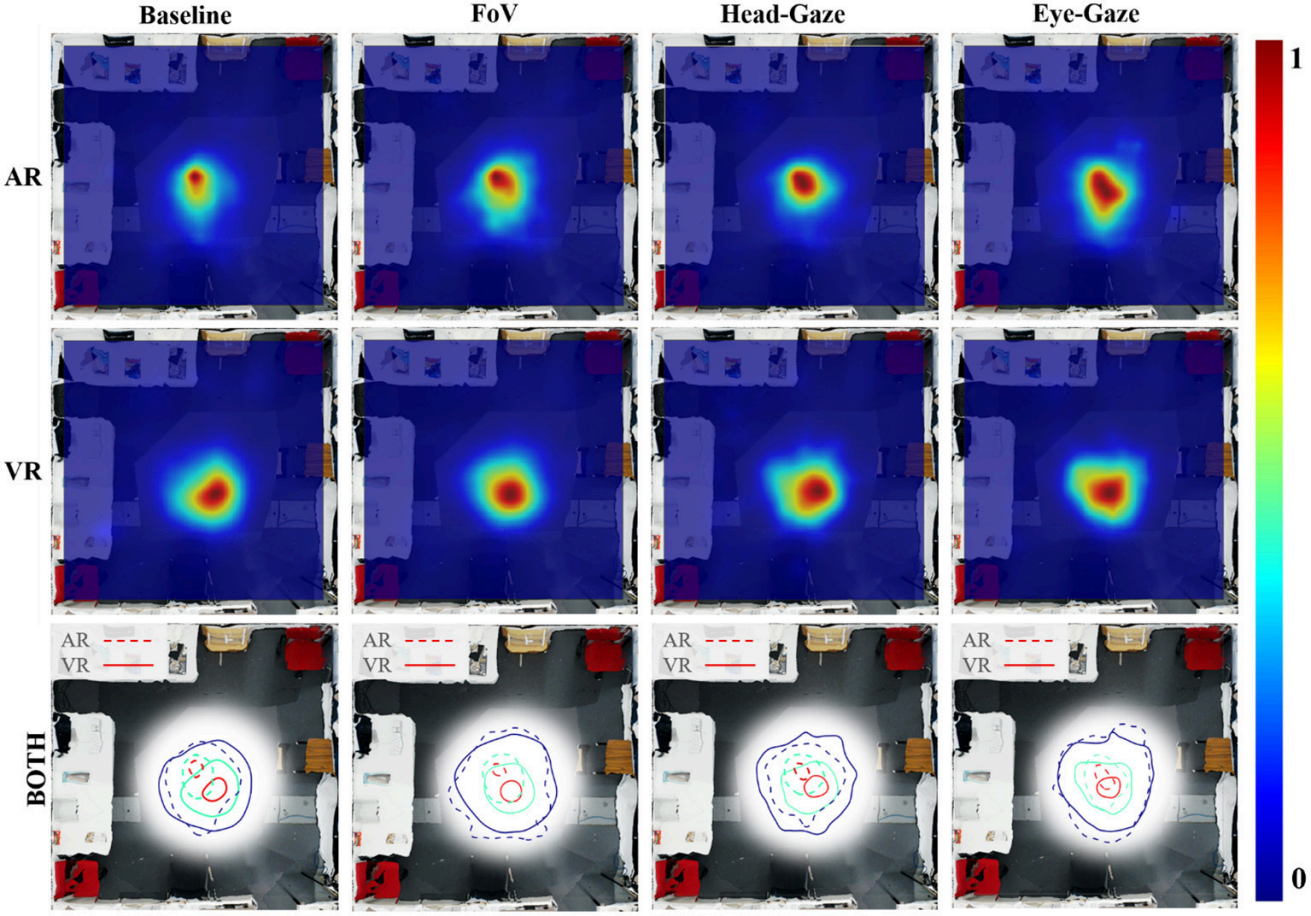
Heat-map of physical movement in the scene by collaborators (Range: 0—less time spent, 1—more time spent): Movement of AR users **(top)**, VR users **(middle)**, and all users using contours with dotted line for AR users and solid line for VR users **(bottom)**.

One major effect of this imbalance can be observed in the user's movement and dwell location. Figure 10 illustrates the user's position heatmaps for both AR and VR users in each condition. It is evident that the VR user was actively and consistently moving around the workspace in all conditions. From the video analysis, we found that most VR users took a proactive role, moving around the scene and leading the AR user to look at the block that they found to be the correct letter. Other evidence supporting this was that VR users performed more pointing gestures than AR users as illustrated in Figure 9. Past research (Steed et al., 1999) had observed similar social behavior in an asymmetric interface, which led to leadership from one side with the argument that embodiment led to more effective gesturing. This characteristic should be examined further and controlled to reduce the effects on the collaboration such that the interfaces do not cause a disparity between collaborator's role unless it is intended.

#### A Good Follower

Another interesting observation was in the Baseline condition, where AR users tended to remain within a smaller area close to the center of the workspace. Again, this is visualized in a heatmap shown in Figure 10. We noticed that the absence of the FoV and gaze cue forced the collaborators to look for each other's head to know their view-direction. In this case, the VR user could easily locate the AR user. To coordinate, the VR user would walk closer to the block and point at it to help the AR user to see it. Some AR users took a passive role in the Baseline condition and waited for the VR users to tell them where to look. This might explain why the positions of the AR users were concentrated in a smaller area at the center of the workspace for the Baseline condition, as it was a good strategic spot to keep track of the VR user and vice versa. Some other AR users stood behind the VR user's shoulder, so they could quickly gaze at the same block and scanned the workspace together systematically.

#### Users' Proximity

By overlaying the AR and VR users' movements in a heatmap, we found that the AR and VR users stood apart from each other and have different peak area, where the users spent the most time in the environment. The Baseline heatmap (Figure 9) showed the two peaks furthest apart followed by the FoV and Head-gaze condition, and they overlapped for the Eye-gaze condition. This coincides with the average distance differences between users that we found where the Baseline condition had the longest distance, followed by the FoV, Head-gaze, and closest being the Eye-gaze condition.

### Effective Verbal Communication

During the study, we did not use any audio equipment or audio cue to enhance the collaboration. Participants spoke with their regular voice and they could hear each other well without the need for microphone and headphone. In pairs of participants who could perform the task effectively, we observed these behaviors from the preliminary video analysis as follows:

#### Thinking Aloud

We found that in some pairs, at least one of the collaborators constantly describing their thoughts or actions. We found that even when the pair were not well-acquainted, this behavior helped the other person understood her/his collaborator better.

#### Initiator

At least one of the two collaborators consistently initiated the conversation. Apart from the visual cues, it was important for someone to initiate the conversation to keep the feeling of co-presence alive and to promote the exchanges of information.

#### Constant Communication

There was a constant communication between them even with short phrases. We encountered a lot of common questions asked between the collaborators. For example, some of the most common questions that VR user asked the AR user were “Can you see my hand?,” “Can you see me?,” and “Can you see where I'm looking?.”

### Limitations

There were a number of limitations in the study that should be addressed in future work. In this section we identify some of the more serious limitations.

#### Factorial Design

We note that the study could have used a factorial design where each visual cue was treated as an individual independent variable. While this was considered at the early stage of planning the user study, we noticed this would increase the number of conditions (16 conditions in total for four factors and each factor with two levels) which would be cumbersome to the participants to try them all. As an alternative, we chose to reduce the combinations of cues to a set which would be most interesting for the purpose of our user study. We plan to further investigate different combinations in future studies.

#### Occasional HMD Shifting

An issue that we encountered on a few occasions during the study was when the HMD shifted from the original eye calibration position due to excessive head movements or the system cables pulling the HMD. This produced errors in the eye-tracking and gaze cue visualization. Even though, the experimenter made sure that the HMD was tightened to the user's head, the shift sometimes occurred. The simple solution without re-calibration was to let the users check by themselves at the reference point such as the center of the screen and manually adjusted and align the eye reticle to the same location. This suggests a research opportunity to better design a well-fitted HMD and to improve the robustness of the eye tracking system.

#### Spatial Audio

The study setup allowed participants to talk to each other physically as in prior works (Gao et al., 2016; Gupta et al., 2016), which could have been improved by using voice over IP software reflecting the typical real-world use of a remote collaboration system. As our study focused on the visual cues, we simply controlled the audio communication to be the same across the conditions. While not included in the scope of the current study, in the future, we plan to employ spatialized audio which could help users understand each other's location purely based on audio cue, and study how audio and visual cues complement each other.

#### Simulated Virtual Tasks

In the study, we used simulated tasks of cooperative search and placement of virtual objects. This was to circumvent having to track physical objects and update their state on the remote VR user's side. However, real-world MR collaboration in the future would likely involve interaction with real-world objects, therefore, future studies will need to take this aspect into consideration when implementing the study's tasks.

## Design Guidelines

From the results and observations of this research, we have compiled the design guidelines for providing virtual awareness cues in collaborative MR as follows:

### Aware and Informed With FoV Frustum

In an asymmetric collaboration between users with different FoV HMDs, FoV frustum can help inform the collaborators of what each user is able to see. Moreover, FoV frustum also assists the user with small FoV to catch a glimpse of the frustum, which helps to indicate the general direction that the collaborator is facing.

### The 3^rd^ Arm With Gaze Ray

Pointing could be performed using our hands. In AR/VR, a raycast from the source's origin to the targeted object helps improve the accuracy of a hand pointing as well as improved shared understanding between collaborators. Normally for precise pointing, users need to look at the target. By taking the advantage of this implicit dependency between gaze and pointing, raycast can be projected from the head or eye gaze instead of an explicit hand pointing. This is possibly useful in the tasks that required bimanual operation leaving no free hand for communication.

### Efficiency Gains Alter Social Behavior

The results and observations showed that virtual awareness cues, FoV frustum, and gaze ray, helped improve the performance on the given tasks in the user study. However, it also altered users' behavior as the tasks emphasized efficiency. We believe that the users should have the freedom to enable or disable the virtual awareness cues to suit the needs of the collaborative tasks. Potentially, with an intelligent user interface, the virtual awareness cues could be shown or hidden by the system, based on the detection of the collaborative context.

## Conclusion and Future Work

In this paper, we presented CoVAR, a novel MR remote collaboration system using AR and VR technology. CoVAR enables an AR user to capture and share the 3D reconstructed local environment with a remote user in VR to collaborate on spatial tasks in a shared space. It supports various interaction methods to enrich collaboration, including gestures, head gaze, and eye gaze input, and provides various awareness cues to improve awareness on remote collaborator's status.

In order to investigate the benefits and effects of different cues on collaboration, we conducted a user study and found objective and subjective evidence of the benefits of having awareness cues to enhance user collaboration. We recommend using FoV frustum to help the users to be aware and informed of their collaborator's attention and possibly use a gaze ray as a third arm to improve communication especially in the tasks that required a bimanual operation. Nevertheless, improving efficiency with these virtual awareness cues might come at a cost of altered users' social behavior in the collaboration, therefore, the users should have a complete control of them.

In the future, we plan to transcribe the discourses in our recordings and conduct an in-depth analysis on it and the other data that we have not included in this paper such as head direction, hand movements. We also plan to further investigate different combinations of visual and audio communication cues while improving the prototype system by adding such features. For the future study that plan to use a combination of sub-tasks such as the identification and placement tasks in this paper; we recommend evaluating the sub-tasks separately, which would yield more definite results.

## Author Contributions

All authors contributed significantly to the conceptualization of this research and the manuscript. TP developed the system with constant input for improvements from MB, BE, GL, and AD. TP and AD conducted the user study and analyzed the data. BE surveyed the related work. All authors contributed to writing the paper.

### Conflict of Interest Statement

The authors declare that the research was conducted in the absence of any commercial or financial relationships that could be construed as a potential conflict of interest.
